# Should deep venous thrombosis prophylaxis be used in fast-track hip and knee replacement?

**DOI:** 10.3109/17453674.2012.672094

**Published:** 2012-04-24

**Authors:** Per Kjærsgaard-Andersen, Henrik Kehlet

**Affiliations:** ^1^Section for Hip and Knee Replacement, Department of Orthopaedics, Vejle Hospital, South Danish University; ^2^Section for Surgical Pathophysiology, Rigshospitalet; ^3^the Lundbeck Centre for Fast-track Hip and Knee Arthroplasty, Copenhagen, Denmark

During the last decade, in-hospital programs for patients undergoing total hip replacement (THA) and total knee replacement (TKA) have changed dramatically from (1) staying in bed for 1–3 days on epidural pain treatment followed by mobilization on crutches for weeks including several restrictions in daily activities, to (2) mobilization a few hours after surgery, with no restrictions in daily activities and no more than 2–4 days in hospital (Kerr and Kohan 2008, Husted et al. 2011, Malviya et al. 2011). This change has been possible due to optimized opioid-sparing multimodal analgesia protocols together with local infiltration analgesia techniques, and a detailed education of the patients before, during, and after the operation.

In that same period, evidence-based guidelines from the American College of Chest Physicians recommended pharmacological prophylaxis after THA and TKA for at least 10 days but preferably up to 35 days (Geerts et al. 2008). This recommendation is mainly based on randomized studies comparing current and new low-molecular-weight heparins (LMWH) and other anticoagulatory agents with a primary efficacy outcome on deep-vein thrombosis (DVT) including the non-symptomatic cases found by venography (Eriksson et al. 2008, Kakkar et al. 2008, Turpie et al. 2009, Lassen et al. 2010a, b). These authors did not state when their patients were mobilized after surgery, or for how many hours a day they were mobilized—factors that are known to have major importance in the development of DVT. However, some of the reports described a hospital stay of between 8 and 12 days (Turpie et al 2009, Lassen et al. 2010a, b), suggesting slow mobilization of patients.

Furthermore, there have been reports of a possible risk of complications when patients are treated with long-term thromboprophylaxis, complications such as wound oozing, bleeding, or deep infection (Jameson et al. 2010). It is therefore important to treat patients for the shortest period possible to prevent symptomatic DVT and pulmonary embolism, but probably not the asymptomatic and more frequent DVT, although the latter requires further evaluation. Orthopedic surgeons worldwide have questioned whether we must treat our joint replacement patients for such long periods as recommended, and there is an open debate on how to create national guidelines for DVT prophylaxis after major joint replacement and other procedures (Davies and Rayment 2010, Polk and Qadan 2010, Treasure et al. 2010, Kakkar and Rushton-Smith 2011, Qadan et al. 2011, Poultsides et al. 2012).

There is an urgent need for randomized studies of today’s fast-track joint replacement patients, to determine whether they really need DVT prophylaxis, and if so, the shortest time required for prophylaxis. Such data might save billions of dollars for our national health systems and prevent patients from being treated for an unnecessarily long time. However, the industry that has supported the current trials (Eriksson et al. 2008, Kakkar et al. 2008, Turpie et al. 2009, Lassen et al. 2010a, b, Lee et al. 2012) will probably neither sponsor nor initiate such studies, for obvious reasons.

In the meantime, we await information from a prospective, large Danish cohort study involving 5,000 fast-track THA and TKA patients with prophylaxis only during the short period in hospital (2–4 days). The results will be analyzed in early 2012. However, preliminary data suggest that there is no need for prolonged prophylaxis in a fast-track setting (Husted et al. 2010) or with early mobilization (Chandrasekaran et al. 2009). Hopefully, international efforts may answer this important question, since the fast-track methodology is becoming more popular (Malviya et al. 2011, McDonald et al. 2011) but with a lack of specific information on thromboprophylaxis regimens despite very low thromboembolic complications (Malviya et al. 2011, McDonald et al. 2011).

In conclusion, there is an urgent need for multicenter studies to assess the requirement for thromboprophylaxis in the context of fast-track THA and TKA.
